# Management of neurobehavioral changes in PSP

**DOI:** 10.1002/alz70857_101938

**Published:** 2025-12-24

**Authors:** Alonso Morales‐Rivero, Raul Medina Rioja, Indira Ruth Garcia Cordero, Carmela Tartaglia, Gabor G. Kovacs, Blas Couto

**Affiliations:** ^1^ Toronto Western Hospital, University Health Network Memory Clinic, Toronto, ON, Canada; ^2^ Instituto Nacional de Neurología y Neurocirugía “Manuel Velasco Suárez”, Mexico, DF, Mexico; ^3^ Universidad de San Andres, Victoria, Buenos Aires, Argentina; ^4^ Division of Neurology, Toronto Western Hospital, University Health Network, Toronto, ON, Canada; ^5^ Rossy PSP Program, University Health Network and the University of Toronto, Toronto, ON, Canada; ^6^ Department of Laboratory Medicine and Pathobiology, University of Toronto, Toronto, ON, Canada; ^7^ Laboratory of Progressive Neuromotor and Neurobehavioral Disorders at Instituto de Neurociencia Cognitiva y Traslacional (INCYT), CABA, Buenos Aires, Argentina; ^8^ Unidad de Movimientos Anormales, Institute of Neuroscience of the Fundacion Favaloro, CABA, Buenos Aires, Argentina

## Abstract

Progressive supranuclear paralysis (PSP) is an atypical parkinsonian disorder associated with oculomotor features, postural instability along with cognitive problems and neuropsychiatric symptoms. Usually, the treatment focuses on motor symptoms, however addressing behavioral and cognitive symptoms could have a significant impact on functionality and quality of life of patients and their relatives. In this session the attendees will learn how to use widely available tools for screening the most common neuropsychiatric symptoms and how to manage them, both with pharmacological treatment and non‐pharmacological interventions. Treatments discussed will include depression, apathy, anxiety, cognitive decline, and other common neuropsychiatric issues. Cognitive impairments in Alzheimer's disease are commonly managed with cholinesterase inhibitors and NMDA receptor antagonists, although their efficacy in non‐Alzheimer's dementias is limited. Behavioral and neuropsychiatric symptoms, including agitation, depression, and apathy, are frequently addressed using antidepressants, antipsychotics, and mood stabilizers, despite the challenges of balancing efficacy with adverse effects. During this session, we will emphasize the importance of comprehensive assessment to better capture the multi‐faceted neurobehavioral impairments seen in PSP patients.

